# Supplementary material for: Neural substrates of cognitive impairment in a NMDAR hypofunction mouse model of schizophrenia and partial rescue by risperidone
Source: Front Cell Neurosci. 2023 Mar 31;17:1152248. doi: 10.3389/fncel.2023.1152248 (PMC10104169; doi:10.3389/fncel.2023.1152248)
Supplement: Supplementary file 1 [file Data_Sheet_1.pdf]

SUPPLEMENTARY INFORMATION FOR

Neural substrates of cognitive impairment in a NMDAR hypofunction mouse model of schizophrenia and partial rescue by risperidone

Cristina Delgado-Sallent, Thomas Gener, Pau Nebot, Cristina López-Cabezón and M. Victoria Puig*

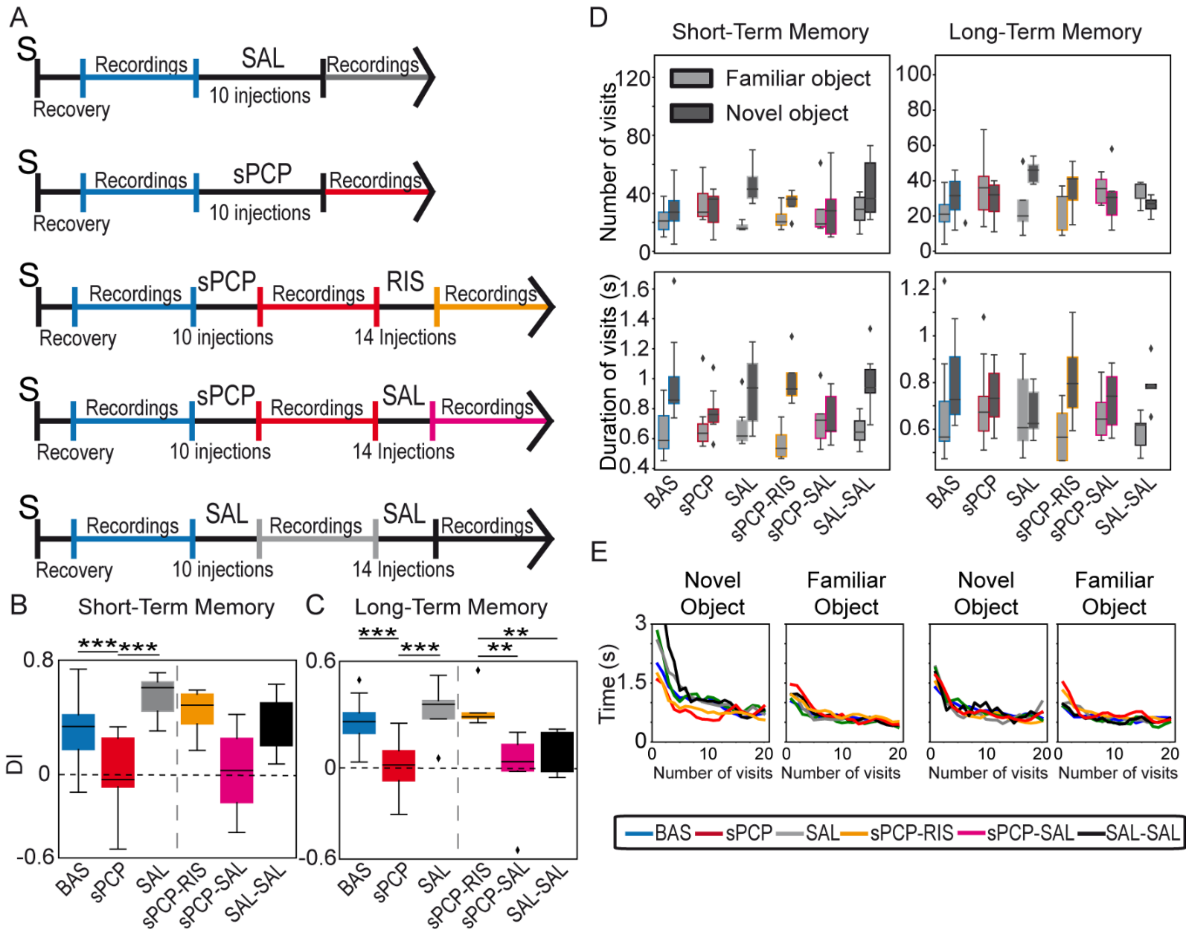

Supplementary Figure 1: (A) Experimental groups of the study: SAL (*n* = 8 mice), sPCP (*n* = 21), sPCP-RIS (*n* = 9), sPCP-SAL (*n* = 7) and SAL-SAL (*n* = 8). The S indicates the day of surgery. **(B)** Discrimination indices of all the pharmacological groups investigated during the 3-minute (short-term) memory test. BAS vs. sPCP vs. SAL: $F_{(1,20)} = 26.31$, $p < 0.0005$, mixed ANOVA. sPCP-RIS vs. sPCP-SAL vs. SAL-SAL: $F_{(2,24)} = 5.11$, $p = 0.017$, two-way ANOVA. **(C)** Discrimination indices of all the pharmacological groups investigated during the 24h (long-term) memory test. BAS vs. sPCP vs. SAL: $F_{(1,26)} = 15.26$, $p = 0.0006$, mixed ANOVA. sPCP-RIS vs. sPCP-SAL vs. SAL-SAL: $F_{(2,24)} = 15.76$, $p < 0.0005$, two-way ANOVA. Vertical dashed lines separate the two statistical groups. **(D)** Number and mean duration of visits to familiar and novel objects during the short-term and long-term memory tests. **(E)** Mean duration of the first 20 visits to familiar and novel objects in the two memory tests.

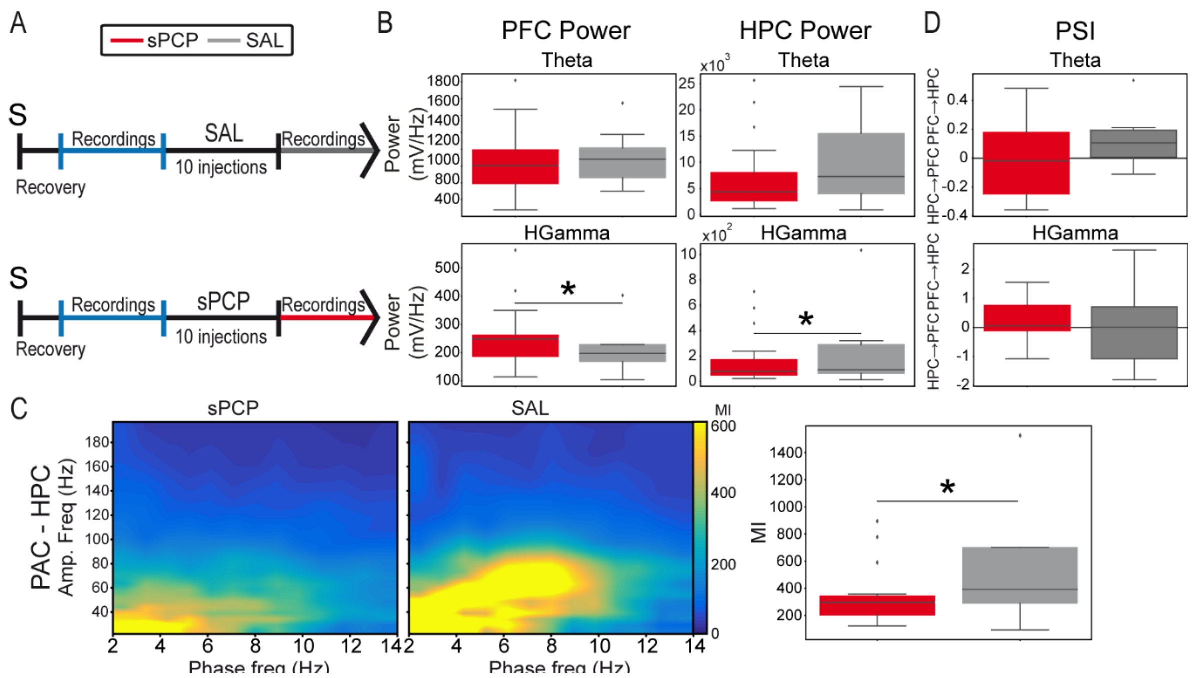

Supplementary Figure 2: Saline controls (SAL group) did not exhibit the alterations observed in the sPCP group during quiet wakefulness. **(A)** Experimental protocols of the sPCP and SAL groups. The S indicates the day of surgery. **(B)** Quantification of theta and high gamma power in both groups. High gamma power increased in the PFC and decreased in the HPC after sPCP but not after saline (sPCP vs. SAL: $F_{(1,22)}$ = 6.59, p = 0.016; two-way ANOVA). **(C)** Local and inter-regional theta-gamma coupling weakened after sPCP but not after saline (sPCP vs. SAL: $F_{(1,22)}$ = 6.31, 4.62, p = 0.02, 0.024; two-way ANOVA). **(D)** The directionality of signals within prefrontal-hippocampal circuits was not affected by sPCP or saline.

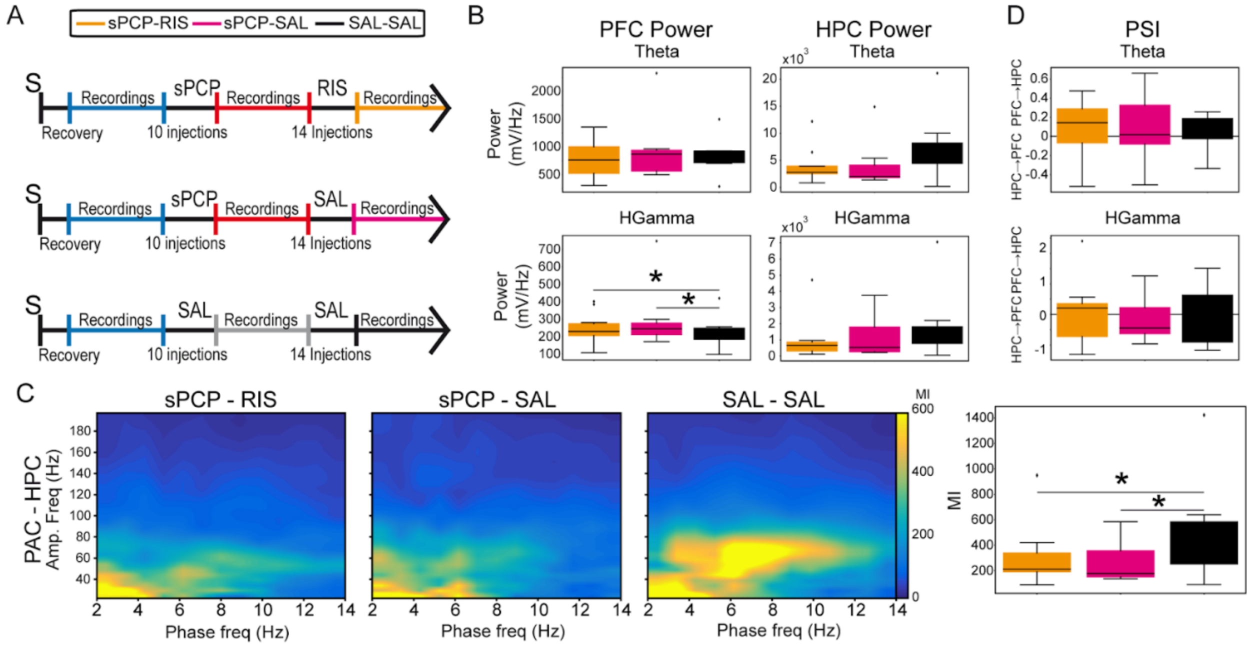

Supplementary Figure 3: Saline controls did not exhibit the alterations observed in the sPCP-RIS group during resting states. **(A)** Experimental protocols of the sPCP-RIS, sPCP-SAL and SAL-SAL groups. S indicates the day of surgery. **(B)** Quantification of theta and high gamma power in the three groups. High gamma power increased in the mPFC and decreased in the dHPC after sPCP, but not after saline. Therefore, in the SAL-SAL group high gamma power was lower in the mPFC and higher in the dHPC (two-way ANOVA) than in the other two groups. **(C)** Local and inter-regional theta-gamma coupling weakened after sPCP but not after saline ([l-PAC, ir-PAC] sPCP-SAL vs. SAL-SAL: $F_{(1,22)}$ = 6.31, 4.62, p = 0.02, 0.024; two-way ANOVA). **(D)** The directionality of signals within prefrontal-hippocampal circuits was similar between groups during quiet alertness states.

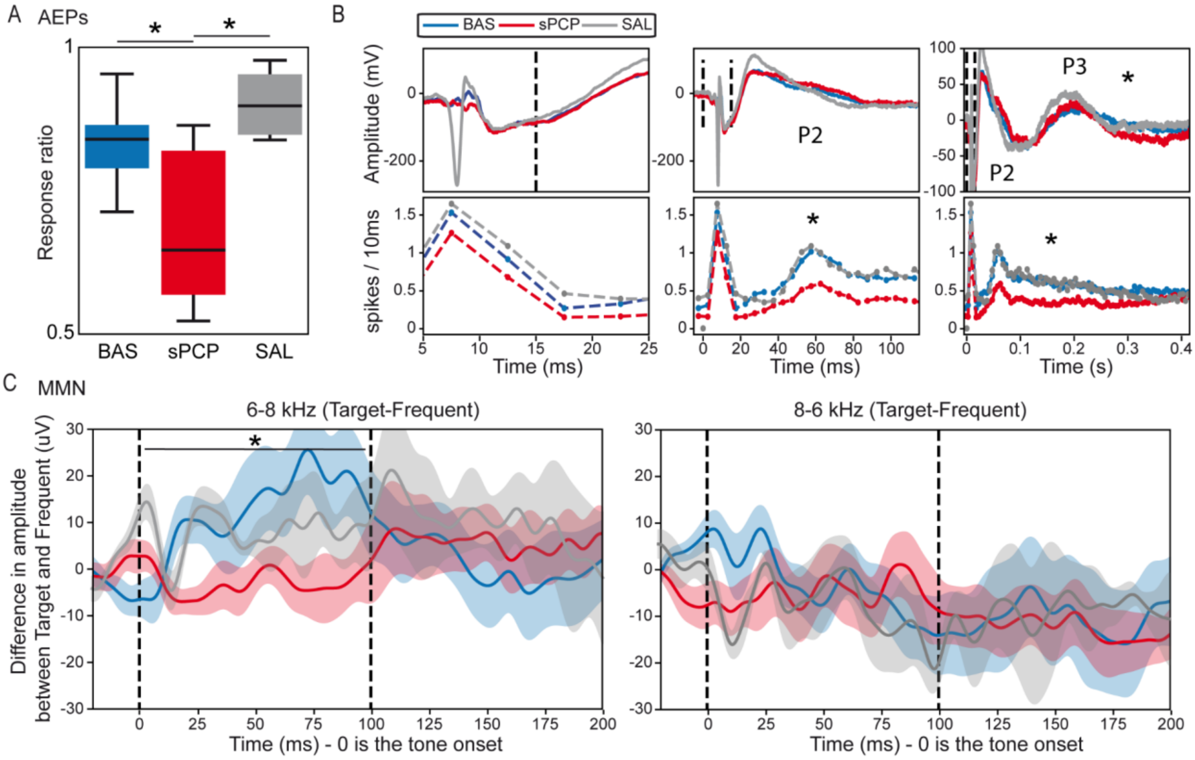

Supplementary Figure 4: After the administration of saline (SAL control group) the behavioural and neurophysiological fingerprints of auditory processing were similar to baseline. **(A)** Ratio of auditory evoked potential (AEPs) responses detected in the mPFC after the presentation of 100 auditory stimuli. The response ratio remained elevated after saline, but not after sPCP. **(B)** AEPs and corresponding spiking activity (multi-unit firing rates) in the mPFC at three different timescales. The neuro-physiological responses were very similar between baseline and after saline. **(C)** Mismatch negativity (MMN) was detected during the presentation of the 6-8KHz target-frequent tone combination. MMN was present in the saline-treated but not the sPCP-treated group. Vertical dashed lines mark the start and end of tone presentation.
